# A profile of traumatic spinal cord injury and medical complications in Latvia

**DOI:** 10.1038/s41394-017-0002-2

**Published:** 2017-12-06

**Authors:** Anda Nulle, Uljana Tjurina, Renars Erts, Anita Vetra

**Affiliations:** 1 National Rehabilitation Centre “Vaivari”, Jurmala, Latvia; 2 Riga Stradins University, Riga, Latvia

## Abstract

**Study design:**

A single centre retrospective study.

**Objectives:**

To collect data and analyse the epidemiological profile of traumatic spinal cord injury and its medical complications during the subacute rehabilitation period.

**Setting:**

Spinal Cord Injury Rehabilitation Programme of the National Rehabilitation Centre, ‘Vaivari’, Jurmala, Latvia.

**Methods:**

Information was collected in 2015 from the medical records of 134 patients with a traumatic spinal cord injury admitted for primary rehabilitation between January 2011 and December 2014.

**Results:**

During this period, the median age of patients with a traumatic spinal cord injury was 39.5 years, and the male to female ratio was 5:1. The leading causes of traumatic spinal cord injuries were falls (37%), road traffic accidents (29%), sport and leisure activities (19%), other cause (8%), unidentified causes (5%), and assault (2%). The most common medical complications were pain (77%), spasticity (48%), urinary tract infections (45%), pressure ulcers (25%), and orthostatic hypotension (14%).

**Conclusions:**

Preventive measures in Latvia should be aimed primarily to address falls, road traffic accidents, and sport and leisure activities in the young male population. Medical complications are varied, and they are an important factor following traumatic spinal cord injury. The results obtained in this study comply with the data from studies in countries of the Baltic and North Sea regions of Europe.

## Introduction

Spinal cord injury (SCI) results in a temporary or permanent loss of motor, sensory, or autonomic functions caudal to the level of damage [1]. The classification of SCI is made in accordance with the International Standards for the Neurologic Classification of SCI and severity of impairment is defined via American Spinal Injury Association Impairment Scale (AIS) [2]. Patients with SCI usually have permanent neurologic deficits and disability.

Medical complications after SCI are common and can affect the patient’s health and the results of the rehabilitation process. The most common complications are pressure ulcers, bladder infections, autonomic dysreflexia and respiratory infections [3, 4]. Medical complications have an adverse impact not only on the patient’s health, but also on social integration, employment probability, and quality of life. Complications may result in death for some groups of patients [5]. The prevention of complications may reduce the economic burden imposed by traumatic spinal cord injury (TSCI), and the application of relevant preventive measures has been shown to avert many of these complications [4].

A better knowledge of TSCI and its medical complications can aid in the design of preventive measures and improve SCI management and treatment outcomes.

There are no available published data concerning TSCI in Latvia.

## Aim of study

The purpose of this study was to collect data and analyse the epidemiological profile of TSCI and its medical complications during the subacute rehabilitation period in Latvia.

## Methods

The study design was retrospective and included data from the medical records of all patients with TSCI who were admitted for the first time to the National Rehabilitation Centre ‘Vaivari’ between January 2011 and December 2014.

The following data were retrieved from the medical history: age, sex, date of trauma, date of admission to the rehabilitation centre, date of discharge, cause of the spinal cord damage and injury level, ASIA classification of the SCI, bladder management on discharge, and medical complications during the rehabilitation course from medical history epicrisis.

Statistical analyses were performed using the IBM SPSS software v. 22.0. P values of less than 0.05 were regarded as statistically significant. Categorical data are presented as a proportion (percentage).

We compared our results with available data from the Baltic and the North Sea regions of Europe.

The study was approved by the Ethical Committee of Riga Stradins University.

## Results

Our report includes 134 patients with TSCI. Among these cases, 42 were registered the year 2011, 31 in 2012, 21 in 2013 and 40 in 2014.

### Age and sex

The mean age of our study sample (*n* = 134) was 41.8 (SD = 15.5 years; range = 17–82).

**Fig. 1:**
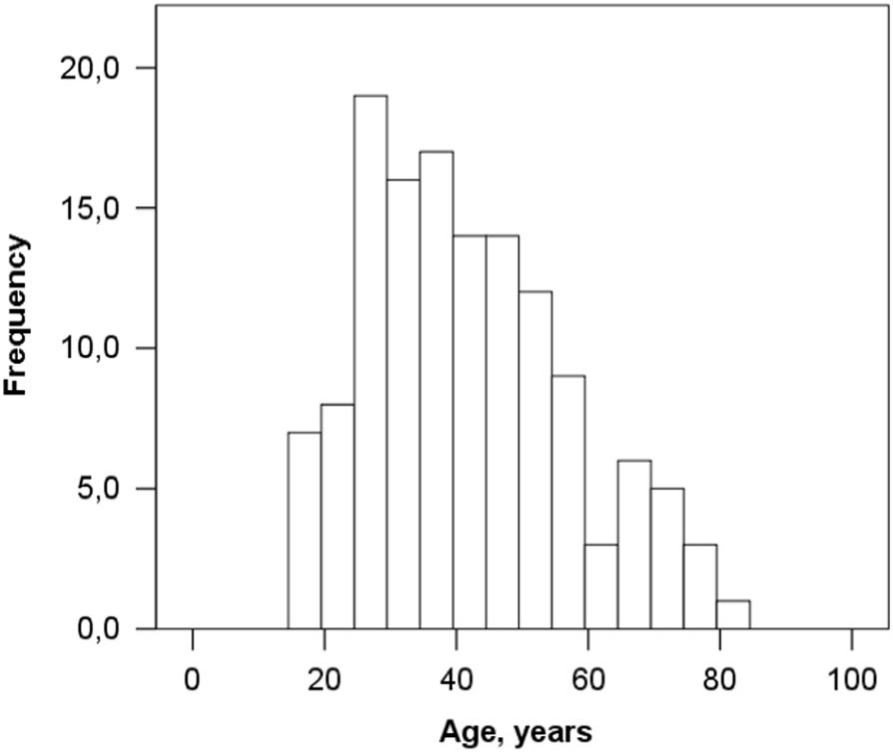
Histogram of patient age

A histogram of the age of the patients is depicted in Fig. 1.

With respect to sex, 122 (84%) of the 134 patients with TSCI were male, and 22 (16%) were female. The male to female ratio was 5:1.

According to the recommendations of the Executive Committee for the Development of the International Spinal Cord Injury Data Sets [6], patients were subdivided into the following age groups: 16–30 years, 31–45 years, 46–60 years and >61 years.

The highest proportion of injuries (48; 36%) occurred in the group of patients aged 31–45. The highest male-to-female injury ratio occurred in patients aged 16–30 (17:1), while the ratio in patients aged 61 and older was 2:1.

**Fig. 2:**
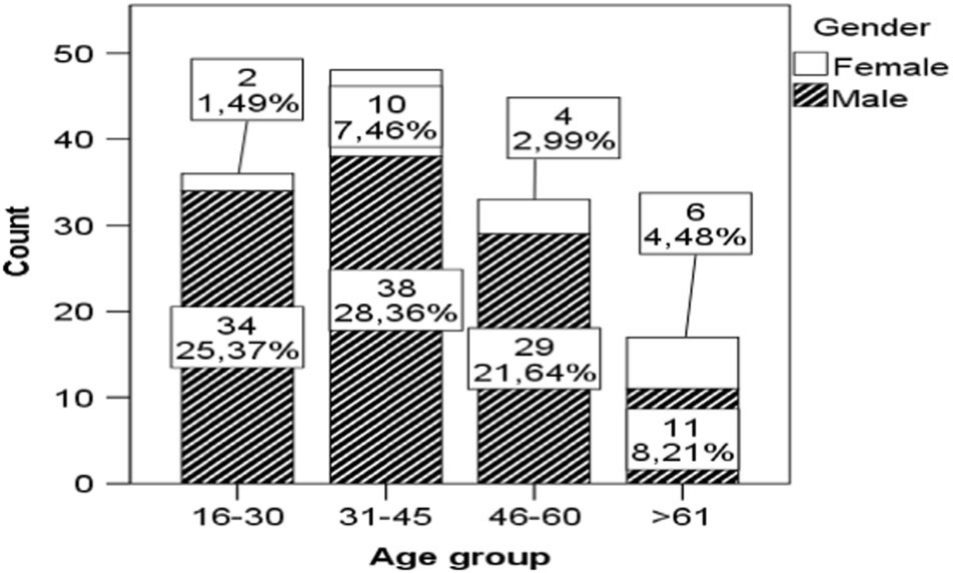
Age and sex distribution

The age and sex distributions are shown in Fig. 2.

### Cause of injury

The causes of TSCI were divided into six groups as suggested by the Executive Committee for the International Spinal Cord Injury Data Sets Committee [6].

**Fig. 3:**
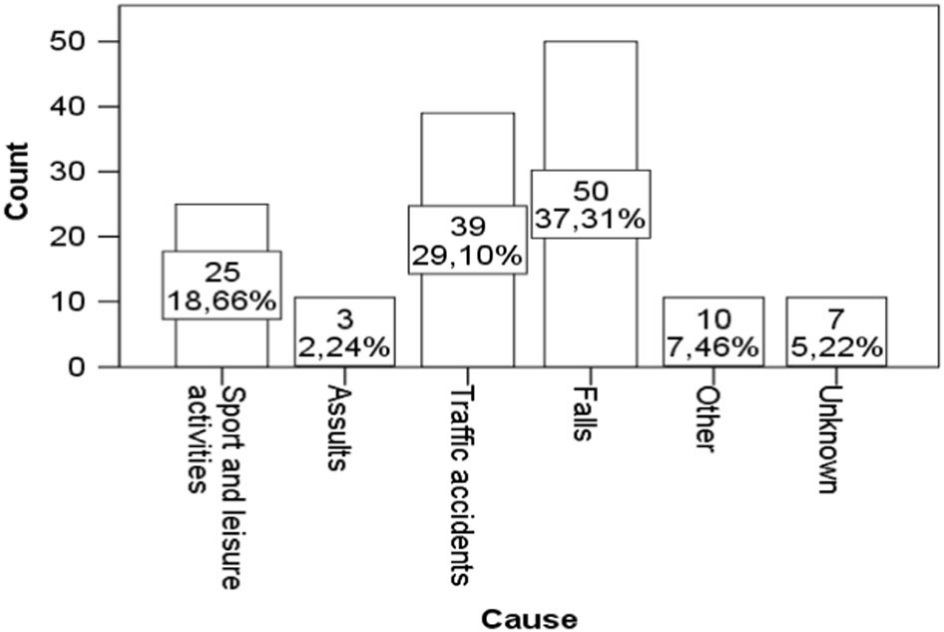
The causes of traumatic spinal cord injury

The leading causes of TSCI were falls (*n* = 50; 37%), road traffic accidents (*n* = 39; 29%), injuries during sports and leisure activities (*n* = 25; 19%), other causes, unknown causes, and assaults. The causes of TSCI are shown in Fig. 3.

**Fig. 4:**
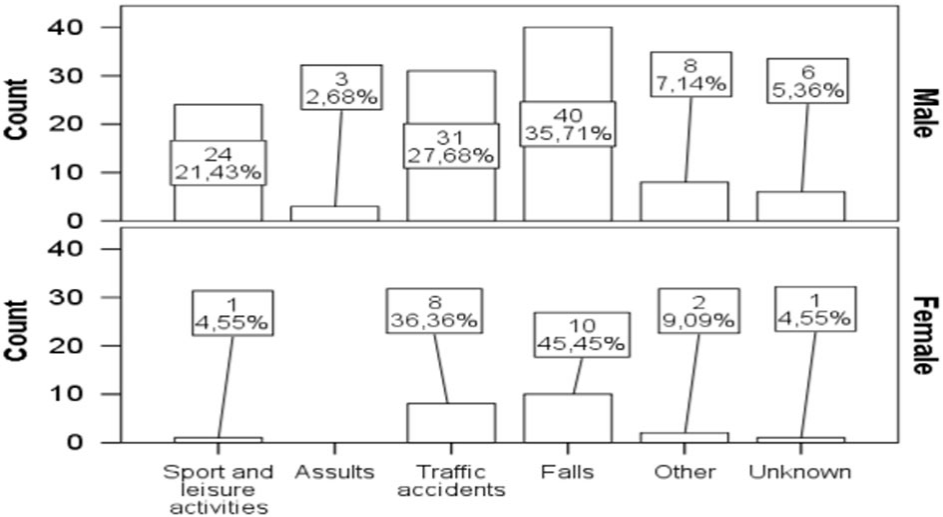
The causes of traumatic spinal cord injury in men and women

The other causes represented 10 cases, in which the patients were injured by a falling tree (*n* = 4), a falling concrete wall (*n* = 1), snow (*n* = 1), a plug (*n* = 1), an explosion (*n* = 1), a suicide attempt (*n* = 1), and an accident involving farming equipment (*n* = 1). The causes of TSCI in men and women are presented in Fig. 4.

The leading causes of injury by age group were as follows: age 16–30 years, sports and leisure activities (14; 41%); age 31–45 years, road traffic accidents and falls (15; 32%); over age 46, falls (16; 49%); and above 61 years, falls (12; 71%) (Fisher’s exact test, *p* < 0.001).

**Fig. 5:**
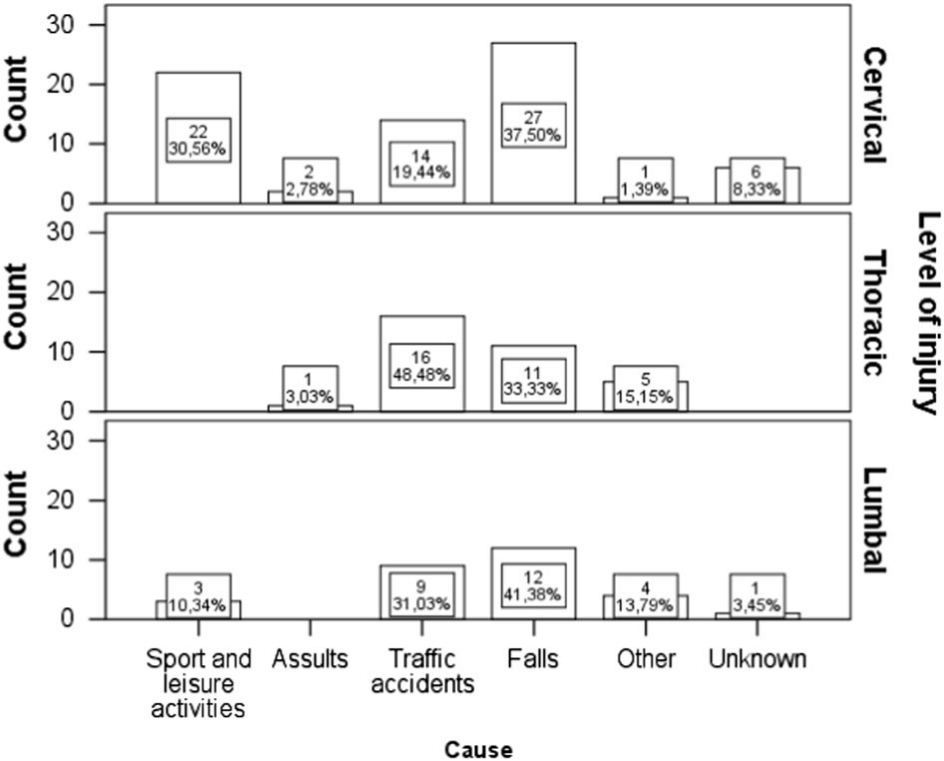
The causes of traumatic spinal cord injury based on level of injury

Injuries to the cervical spine were more common in patients who had trauma due to sports and leisure activities (22; 31%) and falls (27; 38%), while injuries to thoracic area were more often related to road traffic accidents (16; 48%). Injuries to the lumbar area were most often caused by falls (12; 41%) and road traffic accidents (9; 31%) (Fig. 5).

### Level and severity of injury

The traumatic injury occurred in the cervical spine in 72 (54%) of the cases, in the thoracic spine in 33 (25%), and in the lumbar spine in 29 (22%).

The degree of impairment according to AIS was degree A in 35 (26%) of the cases, degree B in 35 (26%), degree C in 25 (19%), D in 36 (27%) and E in 3 (2%).

The mean time between the TSCI and the admission to the rehabilitation centre was 52.4 d (SD = 48.8 d, min = 7 d, max = 304 d).

The mean length of stay was 39.3 d (SD = 19.5 d, min = 5, max = 104 days).

The mean length of stay was 43.9 d for patients with tetraplegia and 36.3 d for paraplegic patients.

Patients with complete tetraplegia had the longest treatment period (62.1 d); in contrast, patients with incomplete paraplegia had the shortest treatment period (30.9 d) (Table 1).

**Table 1:** Time between traumatic spinal cord injury and rehabilitation admission and length of stay

AIS		Time after injury	Length of stay
AISA tetraplegia	Mean	62.13	52.62
	Std. deviation	43.98	12.33
	Minimum	15	37
	Maximum	158	85
AIS A paraplegia	Mean	33.70	44.00
	Std. deviation	22.47	12.64
	Minimum	10	22
	Maximum	92	68
AIS B, C, D tetraplegia	Mean	49.56	41.20
	Std. deviation	44.40	24.33
	Minimum	7	5
	Maximum	200	104
AIS B, C, D paraplegia	Mean	60.42	30.86
	Std. deviation	62.54	12.70
	Minimum	13	11
	Maximum	304	65
Total	Mean	51.97	39.73
	Std. deviation	48.89	19.54
	Minimum	7	5
	Maximum	304	104

*AIS* American Spinal Injury Association Impairment Scale

### Medical complications

The leading complications after traumatic spinal cord injury were as follows: pain in 103 patients (77%), spasticity in 64 (48%), urinary tract infections in 60 (45%), pressure ulcers in 33 (25%), and orthostatic hypotension in 19 (14%). The following complications were reported in selected cases: pneumonia, wound infections, deep vein thrombosis, *Clostridium difficile* colitis, contractures, bladder stones and urinary obstruction, sepsis, autonomic dysreflexia, heterotopic ossification, osteomyelitis and abscess.

Patients with a complete injury are often diagnosed with pressure ulcers and urinary tract infections. Higher-level injuries were associated with the presence of orthostatic hypotension, urinary tract infections and spasticity (Table 2).

**Table 2:** Medical complications associated with traumatic spinal cord injury

Complications/AIS	A	B;C;D	*p*-value	Tetra	Para	*p*-value
Pressure ulcers	19 (54.29%)	14 (14.58%)	< 0.001	20 (28.57%)	13 (21.31%)	0.34
Urinary tract infections	27 (77.14%)	33 (34.38%)	< 0.001	37 (52.86%)	23 (37.70%)	0.08
Pain	29 (82.86%)	71 (73.96%)	0.28	54 (77.14%)	46 (75.41%)	0.81
Spasticity	21 (60%)	43 (44.79%)	0.12	43 (61.43%)	21 (34.43%)	< 0.001
Orthostatic hypotension	8 (22.86%)	11 (11.46%)	0.10	16 (22.86%)	3 (4.92%)	< 0.001

Following TSCI, 75 patients (56%) used intermittent catheterisation for bladder management, 5 patients (4%) used suprapubic indwelling catheterisation, and the remaining 54 patients (40%) had spontaneous voiding. Urinary tract infections were found more frequently in cases of intermittent catheterisation and suprapubic indwelling catheterisation. The number of catheterized patients who had urinary tract infections was 58 patients and only 2 patients had urinary tract infections in cases of spontaneous voiding.

## Discussion

Latvia is one of the Baltic states, with a territory of 64,559 km^2^ and 2 million inhabitants [7].

The Latvian health system provides coverage to the entire population and pays for a basic services package.The National Health Service receives its resources from general tax revenues and purchases care from independent public and private providers [8].

The total healthcare expenditure of Latvia accounted for less than 6.5% of gross domestic product (GDP) and Latvia’s public expenditure on health was 3.7% of its GDP in 2014. Unfortunately Latvia has one of the lowest levels of government spending on health in Europe [9].

The rehabilitation of all TSCI patients is centralized to the National Rehabilitation centre ‘Vaivari’, which provides a specialized inpatient rehabilitation programme for newly injured patients with SCI and a lifelong follow-up programme. The National Rehabilitation Centre ‘Vaivari’ is a state-funded rehabilitation centre with 250 total inpatient beds and 25 beds that are dedicated for patients with SCI. The centre is located in Jurmala, approximately 30 km from the capital city, Riga and is the only place in the nation for subacute SCI rehabilitation; thus, it is representative of the national population. The provision of services is regulated by contracts signed between health care providers from the National Rehabilitation Centre and the National Health Service. The rehabilitation programme for patients with SCI in the post-acute phase was accredited by the Physical and Rehabilitation Medicine section of the European Union of Medical specialists in 2014.

The results of our study show that TSCI is more typical in the young male population, and our data are comparable with those obtained in other countries of the Baltic and the North Sea regions of Europe. The mean age at which an individual sustained an injury, as well as the male-to-female ratio, were similar to that observed in Estonia [10, 11]. The average age of injury in Eastern and Northern European countries ranges from 37–48.9 years. The highest figure has been observed in Western Norway, which has a range of 42.9–48.9 years [11, 12], followed by 47 in Sweden (Stockholm) [13], 38.9–39 in Estonia [10, 11], 38 in Iceland [14] and 37 in Norway [12]. The median age of patients in our study was 39.5 years, and the male to female ratio was 5:1. Estonian statistics show a mean age of 39 and a male to female ratio of 5.5:1 [10, 11]. The highest rate of spinal cord impairments was in individuals below the age of 30 in Denmark, Ireland, and Iceland [14–17], in those aged 16–34 years in Finland [18], and in those aged 20–40 years in Norway [1]. The results of the present study were similar the data obtained in those countries.

In comparing the results on the causes of TSCI, our data were similar to those from Sweden, Western Norway, Estonia and Finland, where falls are the most common cause, followed by road traffic accidents [10–13,18]. The data from Eastern and Northern European countries concerning the level and degree of injury demonstrates a higher prevalence of injuries in the cervical area, similar to the present study. However, a complete impairment was observed slightly less frequently in our study: it was observed in 26.1% of cases, compared to 31–42.3% in the other countries [11, 12, 15, 16].

Knowledge about the medical complications of TSCI is important for designing preventive strategies. Patients with SCI experience pain, spasticity, urinary tract infections, pressure ulcers, and orthostatic hypotension [3, 4], and our study provides additional data. Bladder management with intermittent catheterisation is a safe and efficacious method to treat neurogenic bladder dysfunction due to a spinal cord lesion [19], and 75 (56%) of the patients in our study used this method. We found an association between urinary tract infections and the method of bladder management. Urinary tract infections occurred more often in cases of intermittent catheterisation and suprapubic indwelling catheterisation than in cases of spontaneous voiding. Wyndalee et al. [19] noted that urinary tract infection is the most important complication of intermittent catheterisation, but prevention is possible in the short term. It is important to pay attention to the catheterisation technique and to educate patients. National Rehabilitation Centre’s ‘Vaivari’ clinicians follow the International Guidelines of Bladder management for adults with spinal cord injury [14] and recommendations of International Spinal Cord Society experts [20].

This study was limited by the small number of patients and the clinical data that were collected. This study was the first data collection from patients with TSCI in Latvia. To increase the clinical relevance, more socio-demographic and clinical data should be collected. These data will be helpful for developing a system of care for patients with SCI.

## Conclusion

Primary preventive measures in Latvia should be aimed at the young male population. Following TSCI, medical complications are varied and are an important factor requiring attention throughout rehabilitation. The results obtained in this study comply with the data from studies in other Baltic and North Sea region countries. Future studies should include broader epidemiological data to determine the incidence and provide more clinical statistics on SCI.
